# Diagnostic and management strategies for congenital H-type tracheoesophageal fistula: a systematic review

**DOI:** 10.1007/s00383-020-04853-3

**Published:** 2021-01-20

**Authors:** Keerthika Sampat, Paul D. Losty

**Affiliations:** 1 Department of Paediatric Surgery, Alder Hey Childrens’ Hospital, Liverpool, UK; 2 Institute of Child Health, School of Health And Life Science, University of Liverpool, Liverpool, UK

**Keywords:** H type tracheoesophageal fistula, Congenital oesophageal anomaly, Clinical outcomes, Recurrent laryngeal nerve injury, Newborns, Paediatric

## Abstract

**Background:**

H type tracheoesophageal fistula (H-TEF) is a rare congenital anomaly. Management may be complicated by late diagnosis and variation(s) in the therapeutic strategy. A systematic review of published studies explores the utility of diagnostic studies, operations and postoperative complications.

**Methods:**

Medline and PubMed database(s) were searched for ALL studies reporting H-TEF during 1997–2020. Using PRISMA methodology, manuscripts were screened for eligibility and reporting.

**Results:**

Forty-seven eligible studies were analysed. Primary diagnosis varied widely with surgeons performing oesophagography and trachea-bronchoscopy. Preoperative localisation techniques included fluoroscopy, guidewire placement and catheterisation. A cervical approach (209 of 272 cases), as well as thoracotomy, thoracoscopy and endoscopic fistula ligation, were all described. Morbidity included fistula recurrence (1.7%), leak (2%), tracheomalacia (3.4%) and respiratory sequelae (1%). The major adverse complication in all studies was vocal cord palsy secondary to laryngeal nerve injury (18.5%) yet strikingly few centres routinely reported undertaking vocal cord screening pre or postoperatively.

**Conclusion:**

This study shows that paediatric surgeons record low volume activity with H type tracheoesophageal fistula. Variation(s) in clinical practice are widely evident. Laryngeal nerve injury and its subsequent management warrant special consideration. Care pathways may offset attendant morbidity and define 'best practice.'

**Supplementary Information:**

The online version contains supplementary material available at 10.1007/s00383-020-04853-3.

## Introduction

Congenital tracheoesophageal fistulae are a rare developmental anomaly occurring in around 1 in 2500 live births [1]. The oesophageal malformation and its communication to the respiratory system are categorised into five distinct phenotypes. ‘H-type’, ‘isolated or Gross’ Type E' tracheoesophageal fistula is a very rare subtype accounting for 4–5% of all congenital oesophageal malformations [1]. Patients with H-type fistula may present in the newborn period, infancy, childhood or even adulthood. Operation involving the identification of the fistula tract with the repair of the oesophagus and trachea is curative. Varied strategies are described to precisely localise the fistula and avoid damage to surrounding tissues. A systematic review of published studies was undertaken to explore contemporary surgical practice to benchmark outcomes for this very rare anomaly.

## Methods

A systematic review was conducted in accordance with PRISMA guidelines [2]. A search using MEDLINE and PubMed databases for English articles published since January 1997 was performed. The following search terms were used: H-type fistula, isolated fistula and Gross type E fistula in isolation and association with tracheoesophageal fistula. Additional studies were identified through searching bibliographies and abstracts. The last search was performed in August 2020.

**Fig. 1:**
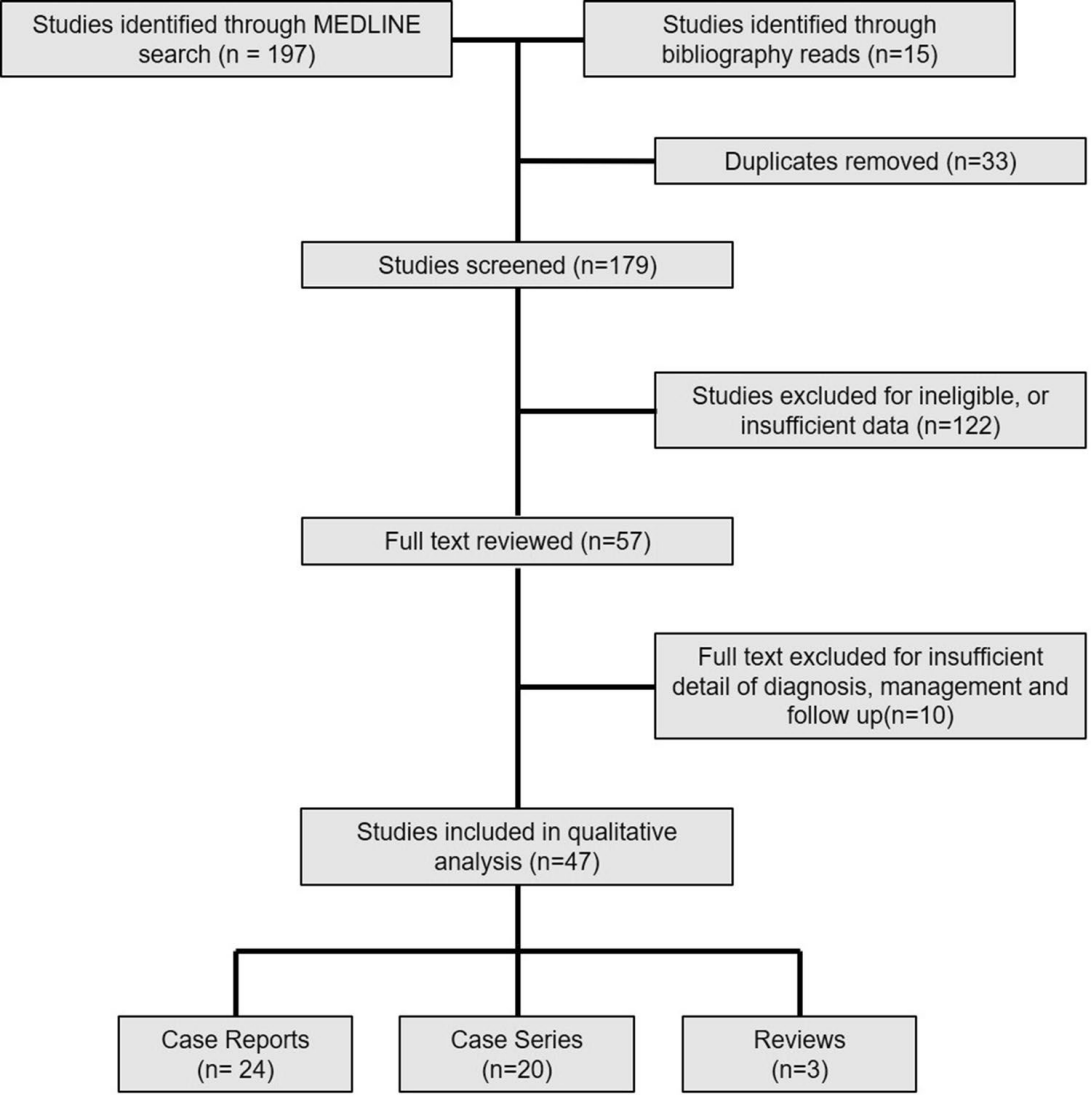
PRISMA flow chart with the literature search strategy

Inclusion criteria were articles reporting human studies on the diagnosis and management of congenital tracheoesophageal fistulae in the absence of oesophageal atresia. Eligible study design(s) included case report(s), case study series and review(s). The article selection process is outlined (Fig. 1). Articles were excluded after scrutinising the title and abstract if the studies were: not in humans and featured acquired or recurrent TEF. Only articles featuring diagnosis and management of patients under 16 years of age were considered, as this group most accurately represents the patient cohort of interest to paediatric surgeons. Full-text articles in which diagnosis, treatment, and follow up of H-type fistula were not discussed were omitted. Any diagnosis or management options relating to H fistula were considered.

Primary and secondary outcomes were extracted in each study. Primary outcomes included: (i) diagnosis, (ii) preoperative fistulae localisation, i.e. strategy deployed, (iii) operative approach and ( iv) reporting of vocal cord injury as an adverse complication. Secondary outcomes were: (i) presenting symptoms, (ii) comorbidities, (iii) other complications and (iv) aftercare follow up.

## Results

### Study description

We identified 197 studies from the search through MEDLINE and an additional 15 reports from bibliography review. Fifty-seven full-text articles were reviewed, and a further ten papers were excluded where insufficient information was given on diagnosis or management. Forty-seven articles featuring a total of 342 patients met the full inclusion criteria. Unsurprisingly, given the rarity of H type tracheoesophageal fistula, most studies were case reports or small case series (< 5 patients) from single institutions. Fallon et al. [3] presented a large multicentre case series, including 102 patients over a 10 year study period [3]. Three reviews were included in the cohort; 2 reviews evaluated open 'classical' cervical vs thoracoscopic and endoscopic approaches and critical report analysis of H-type fistulae from an anaesthetic perspective [4–6].

### Presentation

High rates of co-associated anomalies were reported with H-type fistulae, with Daniel et al. noting 50% of their cohort having another abnormality [5]. The most commonly reported anomalies were cardiac lesions, followed by allied disorders such as VACTERL syndrome (vertebral defects, anal atresia, cardiac defects, tracheoesophageal fistula, renal anomalies, and limb abnormalities) and CHARGE sequence (coloboma, heart defects, atresia choanae, growth retardation, genital abnormalities, and ear abnormalities). The range of comorbidities observed in these studies is highlighted in Table 1.

**Table 1:** Anomalies associated with H-type oesophageal atresia

Associated anomalies (number reported)					
Cardiac	Trisomy	Syndromes	ENT	Gastrointesinal	Other
Unspecified cardiac (29)	Trisomy 21 (5)	VACTERL (7)	Tracheomalacia (3)	Oesophageal reflux (6)	Renal (26)
VSD (12)	Trisomy 18 (1)	CHARGE (4)	Laryngeal cleft (2)	Duodenal atresia (4)	Vertebral (22)
PFO (1)	Trisomy 13 (1)	Other non specified (2)	Choanal atresia (2)	Anorectal malformation (9)	Polydactyly (2)
PDA (4)	Unspecified (5)		Cleft lip and palate (1)	Oesophageal stenosis (4)	Unspecified extra cardiac (4)
Tetralogy of Fallot (4)			Auditory canal atresia (1)	Exomphalos (1)	
Coarctation of aorta (1)					
Hypoplastic arch (1)					

*VSD* ventricular septal defect, *PFO* patent foramen ovale, *PDA* patent ductus arteriosus

In some cases, co-existing pathology aided the prompt diagnosis of H-type fistula. Van Poll et al. cites an example of a vomiting newborn with suspected congenital oesophageal stenosis [7]. In this instance, the contrast swallow study highlighted not only oesophageal stenosis but also fortuitously a bronchogram image revealing an H type fistula. It should be noted that in many patients with life-threatening comorbidities, the subtler symptoms of H-type fistulae may be overlooked entirely. All index patients, regardless of age at diagnosis, will often have occult choking symptoms with feeds and recurrent respiratory infections from early childhood years. The more common presenting symptom in the infant may be linked with periods of apnoea and cyanosis. Although abdominal distension is described as a classic symptom of H-type fistula [1], only a small number of cases from this systematic review were identified and reported. Older patients may suffer for years with suspected gastro-oesophageal reflux. In this review, 3 studies highlighted a delay in diagnosing H-type fistula in patients who had concurrent 'achalasia like symptoms'. In these cases, the H fistula lesion's operative repair dramatically improved symptoms of reflux and ameliorated oesophageal dysmotility [8–10].

### Diagnosis

**Fig. 2:**
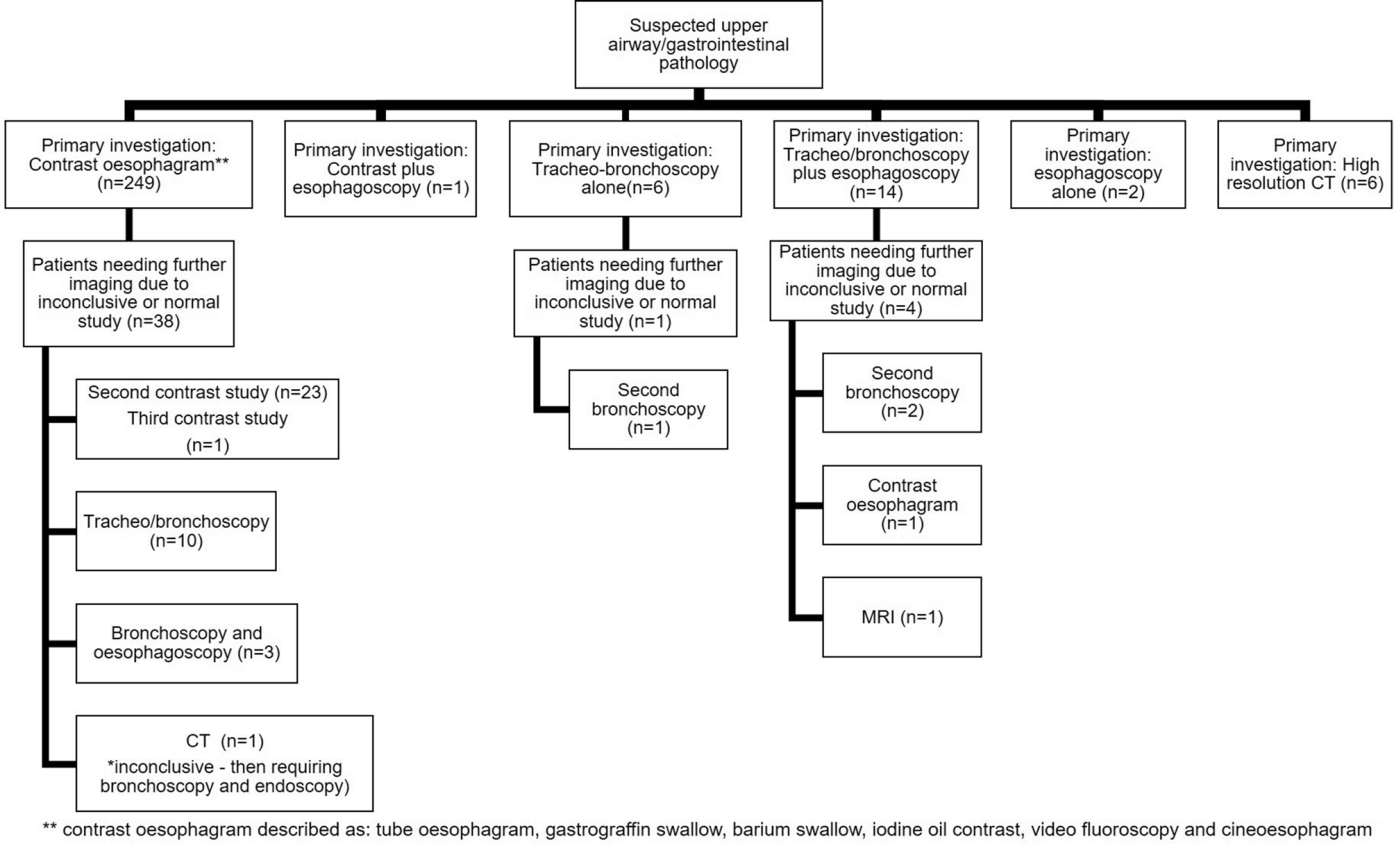
Flowchart of the varied diagnostic pathways for H type oesophageal atresia

Patient age at diagnosis ranged from a few days of life to 15 years. There were no cases of antenatal diagnosis. The majority of index cases may be detected as newborns; Fallon et al. recorded that 69% of patients were diagnosed in the first 30 days of life. Several other published studies show diagnosis at an older age group highlighting the difficulties that may identify this rare pathology. Investigations for H-type fistula aim to confirm the diagnosis and localise the level of the fistula to plan operative repair, e.g. cervical incision vs thoracotomy/thoracoscopy. Diagnostic investigations are broadly divided into radiological modalities such as contrast oesophagram or computed tomography (CT), or endoscopy – such as tracheoscopy. The overwhelming majority of patients underwent a contrast oesophagram as their first line of investigation. It should be noted however that 13% of patients who underwent a primary contrast oesophagram had an inconclusive investigation. Some of these patients went on to have a second and even a third contrast oesophagram confirm the diagnosis [11]. For a minority of patients, where there was a strong suspicion of H-type fistula despite 'reassurance' from a normal contrast swallow examination, a tracheoscopy or bronchoscopy was performed as a second-line investigation. In cases where an endoscopic approach was the first line of investigation but was inconclusive, a combination of tracheo-bronchoscopy and oesophagoscopy, either with or without the use of contrast was reported. Endoscopic studies also yielded false-negative results (2.6%), typically within cases of newborns, where the fistula orifice may be small. Non-invasive diagnostic imaging such as high resolution computed tomography (CT) [9] or magnetic resonance imaging (MRI) [12] have also been utilised in preterm newborns and unstable patients (Fig. 2).

### Operative considerations

Preoperative bronchoscopy and the fistula tract's cannulation were deployed accurately in over 75% of studies we analysed. Cannulation allows precise identification of the fistula tract intraoperatively. Many types of probe devices have been used to cannulate the fistula (Table 2). Steve Rothenberg presents the most extensive single-centre series of thoracoscopic operations. He states visualisation of the recurrent nerve(s) and the H fistula lesion is superior when performed thoracoscopically making cannulation of the tract not always necessary [13].

**Table 2:** Probe devices used to cannulate H fistula pre-operatively

Cannulation method	References
Guidewire	Amat [13], Butterworth [14], De Schutter [15], Sim [16], Antao [17], Allal [18]
Fogarty	Aziz [19], Elebute [20], Riazulhaq [21]
Ureteric stent	Trobs [22]
Ureteric catheter	Ng [8], Garcia [23], Rothenberg [12]
Bronchoscope	Tarcan [24], Goyal [25] we described fibreoptic illumination and entering the fistula with a 2.2 mm scope preoperatively as targeted identification

Identification and operative repair of the H fistula is curative. Many operative approaches are described. The classical right cervical incision with dissection of tissue planes in the root of the neck, isolation and repair of the defect is the most popular. The majority of H fistulae are located above the T2 level, thus allowing optimal access here. In cases of the fistulae lying unusually below T2 level, a thoracotomy or thoracoscopic repair was performed (Table 3).

**Table 3:** Operative approaches to H fistula repair

Approach	*N*
Right cervical	258
Left cervical	22
Anterior cervical	1
Right thoracotomy	10
Thoracoscopic	25
Thoracoscopic converted to open	5
Endoscopic glue	1
Endoscopic YAG laser	1
Electrocautery (converted to open due to oesophageal injury)	3

Majority of studies describe a ligation of the fistula and non-tension closure of both oesophageal and tracheal sides with vicryl or PDS sutures. Al Salem describes a case where the fistula was transfixed only, and in this instance, the fistula reoccurred [11]. Tissue interposition (to offset recurrent fistula) between the oesophagus and trachea was well described in 7 studies. Sternothyroid, subhyoid and strap muscle as well as parietal pleural and fat flaps, are all discussed. Tsai and Zani independently describe deployment of tissue flaps for recurrent fistulae [14, 15]. Insertion of wound drains was reported in only 7 studies. The majority here were small, non-suction devices exiting the cervical incision(s). One chest drain was inserted with a thoracoscopy procedure, and a further single chest drain was placed on suction.

Ten studies, incorporating 16 patients, reported an MIS thoracoscopic approach for H fistula repair. (Table 4) Operative details were however clearly described in only 5 studies. Most surgeons here used a 5 mm telescope. The restricted operative field is often given as a reason not to perform this operation thoracoscopically. Despite this, only two centres used 30-degree scopes, with most utilising a 0-degree scope. The scope position sites were also highly variable, described as being in the anterior and mid-axillary lines, and anterior to the scapula. The time for the operative procedures was quoted as between 50–150 min; however, operation times were not provided in open cervical procedures as a comparator. Times to extubation ranged from 1–5 days and hospital discharge some 2–16 days postoperatively, which is similar to open cases, where reported. Two studies reported complications, notably a tracheal repair failure requiring a second open repair after the initial procedure and diaphragmatic paralysis and vocal cord injury. One thoracoscopic case was converted to an open operation.

**Table 4:** Studies detailing thoracoscopy management of H type fistula

	Number in study	Age at surgery	Level of fistula	Cannulated preoperatively	Telescope details	Operating ports	Port details	Drain	Time (minutes)	Complication	Time to extubation (days)	Time to discharge (days)
Allal [18]	1	8 days	T2–T3	No	5mm 0deg scope mid axillary line 5th intercostal	3mm	3rd and 4th intercostal space in anterior axillary line	Yes	90	None	5	Not reported
Aziz [19]	1	2 months	T2–T3	No	5mm 0deg scope anterior axillary line	2mm	around telescope port	no		None	1	3
Rothenberg [12]	6	2–12 weeks		No	5mm 30deg posterior axillary line	5mm and 3mm	midaxillary line	yes	50	1 Tracheal repair failure	1	2
van Poll [7]	1	6 days		No	5mm 30 degrees. 1 Rib below scapular tip in mid axillary line	3mm	2cm triangulating from telescope port			None		16 *concurrent oesophageal stenosis
Zani [26]	1–No operative details. Converted to open operation repair											
Fallon [3]	1–No operative details											
Lisle [27]	1	8 years	T1	No	5mm anterior axillary line 5th intercostal space	5mm	3rd and 7th intercostal	No	150	None	1	4
Cuestas [28]	1	17 days		Yes-guidewire						Pneumothorax, diaphragmatic paralysis and vocal cord paralysis		
	1	27days		Yes-guidewire							3	
Al Salem [29]	1		‘Low level’	No operative details								

Endoscopic ablation or ligation of H fistula was described in 4 studies. There are various modalities deployed: Cuestas et al. attempted sealing closure of the fistula tract using silver nitrate [16] . However, after 3 recurrences, the fistula was then operatively repaired. Bhatnagar used electrocautery alone in a 13-year-old patient, but again after 2 failed closures, this was corrected at open operation [17]. Tzifa described using electrocautery first, to de-epithelialise the fistula and promote scar formation, followed by applying histocryl glue, which was successful on its first attempt [18]. Of interest, this minimally invasive technique was performed on 2 patients with complete success and no long term complications.

### Postoperative management and complications

The majority of patients in all reported studies were extubated within 48 h postoperatively (range 1 day–5 weeks). An oral contrast study was undertaken to exclude leak or recurrence of fistula in most but not all patients. Fallon reported that from some 102 patients, 78% had an oral contrast examination on day 7 postoperatively. Of that group, 8% ( 5% of the total cohort) were found to have a leak. Sim and Kane both reported a second fistula's discovery which went on to require further corrective surgery [19, 20]. Stenosis of the oesophagus, either as a primary pathology or secondary to fistula repair was also noted and oesophageal dilatation(s) undertaken in symptomatic patients. [21] Rates of fistula recurrence in these patients were low, between 2–3% of all cases reported. Mortality after H fistula operation in all reported studies was low. This occurred was often linked to other co-existent pathologies such as structural cardiac malformations or extreme prematurity. We observed 5 deaths (1.7%) in all eligible reviewed studies [3, 12, 22].

The duration of hospital inpatient stay varied considerably depending on comorbidity(s) or other complications. Where no other pre-existent conditions prevailed, inpatient stay was typically between 2–10 days.

The most frequently cited complication(s) with H fistula's operative repair was vocal cord injury and gastro-oesophageal reflux. Vocal cord dysfunction may be typically unilateral or bilateral with patients having pronounced hoarseness, feeding difficulty and recurrent pneumonia(s). Fallon et al. strikingly reported that some 22% of patients had vocal cord injury [3]. However, only one-third of all patients here had formal postoperative laryngoscopy examinations with vocal cord assessment, and so morbidity may be much higher than that reported. Fung and Zani both observed 50% vocal cord dysfunction rates in their studies [15, 23]. There is little data regarding congenital vocal cord paralysis. Indeed, there is a case to be made for preoperative assessment of function to reflect iatrogenic operative injury rates accurately [24]. From the eligible publications we scrutinised, the majority of patients showed degrees of resolution without intervention. Tracheostomies were undertaken and reported in 2 studies to provide patients with a safe patent airway [3, 25].

Gastroesophageal reflux may be observed in patients with co-existent H type fistula, and in some cases, this alone may lead to a delay in primary diagnosis. Here of interest, a fundoplication was performed in 25 (8.7%) patients with H fistula. Timing of anti-reflux surgery was variably reported with some fundoplication operations undertaken before H fistula repair then leading to the 'late' diagnosis of the anomaly and others repaired concurrently.

Aftercare follow up visits after H-TEF repair where reported ranged from 3 months—9 years.

## Discussion

H type tracheoesophageal fistula without oesophageal atresia is a rare entity seen in 1 in 50,000 to 80,000 live births and accounts for 4–5% of all congenital oesophageal anomalies [1]. Patients with this rare condition may not always be diagnosed in the immediate newborn period. Late diagnosis may occur as the newborn can feed with subtle airway aspiration features, and recurring chest infections may be erroneously attributed to gastro-oesophageal reflux. In this systematic review of all eligible published studies, it was apparent that the well-described triad of classic symptoms notably—abdominal distension, respiratory infection(s), choking and cyanosis on feeding, are not always specific enough to lead to early primary diagnosis. Delay in diagnosis may portend severe morbidities, like that reported in the 15-year-old patient described by Boybeyi et al. This late case presented with irreversible lung damage after years of occult aspiration and chest infection(s) [9]. Late or missed diagnosis may also be due ( in part) to the variable anatomy of the fistula. A small and narrow fistula tract may be overlooked on the initial investigation(s), especially in older children where the oesophagus' mucosal folds may act intermittently as a ‘protective valve’ [30]. Thirteen per cent of all primary investigations were falsely negative in this comprehensive systematic review of the literature. There is no single investigation with sufficient sensitivity and specificity to aid ready accurate diagnosis of H-type fistula. However, a diagnostic algorithm which may improve detection rates should include a dynamic prone contrast oesophagram along with a tracheo-bronchoscopy study for precise localisation [26]. Non-invasive imaging, notably CT and MRI, are perhaps best reserved for the extreme preterm newborn where small patient size precludes airway instrumentation and endoscopy.

Preoperative, awake tracheo-bronchoscopy as a diagnostic tool allows ready identification of the fistula, assessing vocal cord motility, and evaluating other concurrent pathology, such as tracheomalacia [3]. Airway endoscopy facilitates fistula tract cannulation and its bright field 'illumination ' guiding the surgeon intraoperatively [27].

Identifying the level of the fistula is crucial as this dictate operative approach. Three-quarters of isolated H fistulae are located at the T2 vertebra level or above [4]. Here a trans-cervical approach offers optimal access. Genty has outlined that a surgeon's preference may dictate their clinical practice: with paediatric surgeons often employing a right cervical incision vs a left cervical incision used by the ENT specialist [29]. Fistulae located below the level of T2 are most commonly accessed either open via a thoracotomy or thoracoscopy [28] Enthusiasts for thoracoscopic repair argue that there is wholly adequate visualisation and access to the H fistula lesion without the morbidity associated with thoracotomy such as winging of the scapulae or scoliosis. In a systematic review of thoracoscopy cases, Parolini et al. described a handful of case report(s) and cohort series' with considerable variability in patient positioning, instrumentation, operating time(s) and postoperative recovery [4] There remains only a few case series' of thoracoscopic cases. So, whether there is a real advantage in utilising thoracoscopy over an open approach remains open to debate. However, as surgeons' numbers using a thoracoscopic approach increases, new, more robust data may guide future practice. Endoscopic closure of H fistulae has been reported with varied techniques such as tissue glue adhesives, electrocautery, sclerosant agents and laser ablation. Although these may be theoretically appealing as much less invasive methods, there appears to be higher rate(s) of fistula recurrence in these published studies [5].

The most commonly reported associated morbidity(s) with H fistula operation are gastroesophageal reflux and vocal cord injury. Although dysphagia and reflux are much more frequently seen in patients with other variant lesions within the spectrum of oesophageal atresia, a cohort of patients with isolated H type fistula experienced medically resistant reflux underwent anti-reflux surgery [1] Lemoine describes intrinsic oesophageal dysmotility in patients with H fistulae before undergoing operative correction [31] In this systematic review, we found three recorded H fistula cases associated with congenital oesophageal stenosis and achalasia symptoms. This suggests that although the H fistula lesion may appear to be the predominant anatomical defect, there is likely a spectrum of variant pathology observed with oesophageal anomalies and H-type fistula.

Vocal cord injury (up to 18.5% index cases) is a major adverse complication after H fistula repair. It may present as an acute or chronic airway problem in the absence of fistula recurrence. For some patients, vocal cord injury may lead to unsafe swallow necessitating modification of feeds, or use of feeding tubes. Most significantly bilateral vocal cord injury may lead to a compromised airway, requiring definitive management with a tracheostomy. Despite being recognised as a not infrequent and a serious complication associated with H-TEF repair, very few institutions routinely screen for vocal cord injury postoperatively. Zani et al. reported that while such symptoms may resolve, it is concerning that only one-third of all patients with vocal cord injury after H fistula operation regained normal vocal cord function in aftercare follow up. The Toronto group now advocate a multidisciplinary care team to include ENT surgeons in the long term follow-up and monitoring of these patients [15]. Parents should, therefore, always receive accurate preoperative counselling by the operating surgeon detailing the risk(s) of recurrent laryngeal nerve injury together with fistula recurrence when scheduling operation.

We fully acknowledge that this systematic review study has limitations. The eligible studies were all retrospective and for the most part cohort series or case report(s), reflective of the rare incidence of H type tracheo-oesophageal fistulae. Also, there is a variation in the diagnostic, management and follow up outcomes reported. Nonetheless, we fully report the utility of various diagnostic studies, care pathway algorithms and outcome metrics from the quality of the published literature available.

In closing, we believe this study helpfully provides paediatric surgeons with collective informative metrics allowing them to benchmark their own clinical outcome(s) and performance when operating on patients with rare H type tracheoesophageal anomalies.

## Supplementary Information

Below is the link to the electronic supplementary material.

Supplementary file1 (DOCX 46 KB)
