# Supplementary material for: Diagnostic and management strategies for congenital H-type tracheoesophageal fistula: a systematic review
Source: Pediatr Surg Int. 2021 Jan 20;37(5):539–47. doi: 10.1007/s00383-020-04853-3 (PMC8026411; doi:10.1007/s00383-020-04853-3)
Supplement: Supplementary file 1 — Supplementary file1 (DOCX 46 KB) [file 383_2020_4853_MOESM1_ESM.docx]

Appendix 1

All articles included in review

(1–15)(16–35)(36)(37–47)

1. Allal H, Montes-Tapia F, Andina G, Bigorre M, Lopez M, Galifer RB. Thoracoscopic repair of H-type tracheoesophageal fistula in the newborn: A technical case report. J Pediatr Surg. 2004;39(10):1568–70.

2. Al-Salem AH, Mohaidly M Al, Al-Buainain HMH, Al-jadaan S, Raboei E. Congenital H-type tracheoesophageal fistula: a national multicenter study. Pediatr Surg Int. 2016;32(5):487–91.

3. Boybeyi Ö, Köse M, Ersöz DD, Haliloglu M, Karnak I, Şenocak ME. Achalasia-like findings in a case with delayed diagnosis of H-type tracheoesophageal fistula. Pediatr Surg Int. 2008;24(8):965–9.

4. Brookes JT, Smith MC, Smith RJH, Bauman NM, Manaligod JM, Sandler AD. H-type congenital tracheoesophageal fistula: University of Iowa experience 1985 to 2005. Ann Otol Rhinol Laryngol. 2007;116(5):363–8.

5. Butterworth SA, Webber EM, Jamieson DH. Pediatric surgical images: H-type tracheoesophageal fistula. J Pediatr Surg. 2001;36(6):958–9.

6. Conforti A, Iacusso C, Valfrè L, Trozzi M, Bottero S, Bagolan P. Cervical repair of congenital tracheoesophageal fistula: Complications lurking! J Pediatr Surg [Internet]. 2016;51(10):1623–6. Available from: http://dx.doi.org/10.1016/j.jpedsurg.2016.06.010

7. Cuestas G, Rodríguez V, Millán C, Munzón PB, Munzón GB. H-type tracheoesophageal fistula in the neonatal period: Difficulties in diagnosis and different treatment approaches. A case series. Arch Argent Pediatr. 2020;118(1):56–60.

8. Amat F, Heraud MC, Scheye T, Canavese M, Labbé A. Flexible bronchoscopic cannulation of an isolated H-type tracheoesophageal fistula in a newborn. J Pediatr Surg [Internet]. 2012;47(10):e9. Available from: http://dx.doi.org/10.1016/j.jpedsurg.2012.05.012

9. Antabak A, Luetic T, Caleta D, Romic I. H-type Tracheoesophageal Fistula in a Newborn: Determining the Exact Position of Fistula by Intra-operative Guidewire Placement. J neonatal Surg. 2014;3(3):36.

10. Antao B, Soccorso G, Bateman N, Shawis R. H-type tracheoesophageal fistula with type III laryngotracheoesophageal cleft. Eur Arch Oto-Rhino-Laryngology. 2007;264(11):1373–6.

11. Jaiswal AA, Garg AK, Mohanty MK. “H” type tracheo-oesophageal fistula - Case reports with review of the literature. Egypt J Ear, Nose, Throat Allied Sci [Internet]. 2014;15(2):143–8. Available from: http://dx.doi.org/10.1016/j.ejenta.2013.12.007

12. Aziz GA, Schier F. Thoracoscopic ligation of a tracheoesophageal H-type fistula in a newborn. J Pediatr Surg. 2005;40(6):35–6.

13. Babu R, Hutton KAR, Spitz L. H-type tracheo-oesophageal fistula with congenital oesophageal stenosis. Pediatr Surg Int. 2005;21(5):386–7.

14. Batra YK, Rajeev S, Rao KLN. Severe stridor following the repair of H-type tracheoesophageal fistula in an 11 month infant. Acta Anaesthesiol Belg. 2008;59(4):267–71.

15. Bhatnagar V, Lal R, Sriniwas M, Agarwala S, Mitra DK. Endoscopic treatment of tracheoesophageal fistula using electrocautery and the Nd:YAG laser. J Pediatr Surg [Internet]. 1999 Mar [cited 2020 May 11];34(3):464–7. Available from: http://www.ncbi.nlm.nih.gov/pubmed/10211655

16. Dai J, Pan Z, Wang Q, Wu Y, Wang J, Wang G, et al. Experience of diagnosis and treatment of 31 H-type tracheoesophageal fistula in a single clinical center. Pediatr Surg Int [Internet]. 2018;34(7):715–9. Available from: http://dx.doi.org/10.1007/s00383-018-4293-6

17. Daniel SJ, Smith MM. Tracheoesophageal fistula: Open versus endoscopic repair. Curr Opin Otolaryngol Head Neck Surg. 2016;24(6):510–5.

18. Goyal A, Potter F, Losty PD. Transillumination of H-type tracheoesophageal fistula using flexible miniature bronchoscopy: An innovative technique for operative localization. J Pediatr Surg. 2005;40(6):33–4.

19. Gunlemez A, Anik Y, Elemen L, Tugay M, Gökalp AS. H-type tracheoesophageal fistula in an extremely low birth weight premature neonate: Appearance on magnetic resonance imaging. J Perinatol. 2009;29(5):393–5.

20. Sim J, Hong J. Double H-type Tracheoesophageal Fistulae: A Case Report. Adv Pediatr Surg. 2018;24(2):94.

21. Kane TD, Atri P, Potoka DA. Triple fistula: management of a double tracheoesophageal fistula with a third H-type proximal fistula. J Pediatr Surg. 2007;42(6):10–2.

22. Karnak I, Şenocak ME, Hiçsönmez A, Büyükpamukçu N. The diagnosis and treatment of H-type tracheoesophageal fistula. J Pediatr Surg. 1997;

23. Ko A, DiTirro FR, Glatleider PA, Applebaum H. Simplified access for division of the low cervical/high thoracic H-type tracheoesophageal fistula. J Pediatr Surg. 2000;35(11):1621–2.

24. Lastinger A, El Yaman M, Gustafson R, Yossuck P. Scimitar Syndrome and H-type Tracheo-esophageal Fistula in a Newborn Infant. Pediatr Neonatol [Internet]. 2016;57(3):236–9. Available from: http://dx.doi.org/10.1016/j.pedneo.2013.06.010

25. Le SDV, Lam WWM, Tam PKH, Cheng W, Chan FL. H-type tracheo-oesophageal fistula: Appearance on three-dimensional computed tomography and virtual bronchoscopy. Pediatr Surg Int. 2001;17(8):642–3.

26. Lisle RM, Nataraja RM, Mahomed AA. Technical aspects of the thoracoscopic repair of a late presenting congenital H-type fistula. Pediatr Surg Int. 2010;26(12):1233–6.

27. Mattei P. Double H-type tracheoesophageal fistulas identified and repaired in 1 operation. J Pediatr Surg [Internet]. 2012;47(11):e11–3. Available from: http://dx.doi.org/10.1016/j.jpedsurg.2012.06.012

28. De Schutter I, Vermeulen F, De Wachter E, Ernst C, Malfroot A. Isolated tracheoesophageal fistula in a 10-year-old girl. Eur J Pediatr. 2007;166(9):911–4.

29. Donnelly P, McVea S, Flannigan C, Bali S. Incidental diagnosis of an H-type tracheo-oesophageal fistula. BMJ Case Rep. 2016;2016:1–4.

30. Edelman B, Selvaraj BJ, Joshi M, Patil U, Yarmush J. Anesthesia Practice: Review of Perioperative Management of H-Type Tracheoesophageal Fistula. Anesthesiol Res Pract. 2019;2019.

31. Elebute OA, Ademuyiwa AO, Seyi-Olajide JO, Bode CO. H-type tracheo-oesophageal fistula: a diagnostic challenge in a resource-poor country. A case report. Niger Postgrad Med J [Internet]. 2013 Sep [cited 2020 May 11];20(3):234–6. Available from: http://www.ncbi.nlm.nih.gov/pubmed/24287758

32. Fallon SC, Langer JC, St. Peter SD, Tsao KJ, Kellagher CM, Lal DR, et al. Congenital H-type tracheoesophageal fistula: A multicenter review of outcomes in a rare disease. J Pediatr Surg [Internet]. 2017;52(11):1711–4. Available from: https://doi.org/10.1016/j.jpedsurg.2017.05.002

33. Fung SW, Lapidus-Krol E, Chiang M, Fallon EM, Haliburton B, Propst EJ, et al. Vocal cord dysfunction following esophageal atresia and tracheoesophageal fistula (EA/TEF) repair. J Pediatr Surg. 2019;54(8):1551–6.

34. Garcia NM, Thompson JW, Shaul DB. Definitive localization of isolated tracheoesophageal fistula using bronchoscopy and esophagoscopy for guide wire placement. J Pediatr Surg. 1998;33(11):1645–7.

35. Genty E, Attal P, Nicollas R, Roger G, Triglia JM, Garabedian EN, et al. Congenital tracheoesophageal fistula without esophageal atresia. Int J Pediatr Otorhinolaryngol. 1999;48(3):231–8.

36. Holcomb GW, Rothenberg SS, Bax KMA, Martinez-Ferro M, Albanese CT, Ostlie DJ, et al. Thoracoscopic repair of esophageal atresia and tracheoesophageal fistula: A multi-institutional analysis. In: Annals of Surgery. 2005.

37. Ng J, Bartram J, Antao B, Everard M, Shawis R. H-type tracheoesophageal fistula masquerading as achalasia cardia in a 13-year-old child. J Paediatr Child Health. 2006;42(4):215–6.

38. Parolini F, Morandi A, Macchini F, Gentilino V, Zanini A, Leva E. Cervical/thoracotomic/thoracoscopic approaches for H-type congenital tracheo-esophageal fistula: A systematic review. Int J Pediatr Otorhinolaryngol [Internet]. 2014;78(7):985–9. Available from: http://dx.doi.org/10.1016/j.ijporl.2014.04.011

39. Zani A, Jamal L, Cobellis G, Wolinska JM, Fung S, Propst EJ, et al. Long-term outcomes following H-type tracheoesophageal fistula repair in infants. Pediatr Surg Int. 2017;33(2):187–90.

40. Riazulhaq M, Elhassan E. Early recognition of h-type tracheoesophageal fistula. APSP J Case Rep [Internet]. 2012;3(1):4. Available from: http://www.ncbi.nlm.nih.gov/pubmed/22953298%0Ahttp://www.pubmedcentral.nih.gov/articlerender.fcgi?artid=PMC3418037

41. Rothenberg SS. Thoracoscopic management of non-type C esophageal atresia and tracheoesophageal atresia. J Pediatr Surg [Internet]. 2018;53(1):121–5. Available from: https://doi.org/10.1016/j.jpedsurg.2017.10.025

42. Singh S, Wakhlu A. Megaesophagus in the pediatric age group: A diagnostic dilemma. Saudi J Gastroenterol. 2012;18(2):151–2.

43. Tarcan A, Gürakan B, Arda S, Boybat F. Congenital H-type fistula: Delayed diagnosis in a preterm infant. J Matern Neonatal Med. 2003;13(4):279–80.

44. Tröbs RB, Finke W, Bahr M, Roll C, Nissen M, Vahdad MR, et al. Isolated tracheoesophageal fistula versus esophageal atresia – Early morbidity and short-term outcome. A single institution series. Int J Pediatr Otorhinolaryngol. 2017;94:104–11.

45. Tzifa KT, Maxwell EL, Chait P, James AL, Forte V, Ein SH, et al. Endoscopic treatment of congenital H-Type and recurrent tracheoesophageal fistula with electrocautery and histoacryl glue. Int J Pediatr Otorhinolaryngol. 2006;70(5):925–30.

46. Van Poll D, Van Der Zee DC. Thoracoscopic treatment of congenital esophageal stenosis in combination with H-type tracheoesophageal fistula. J Pediatr Surg [Internet]. 2012;47(8):1611–3. Available from: http://dx.doi.org/10.1016/j.jpedsurg.2012.05.015

47. Wright TN, Grant C, Hirschl RB, Lal DR, Minneci PC, Fallat ME. Neural monitoring during H-type tracheoesophageal fistula division: A way to decrease recurrent laryngeal nerve injury? J Pediatr Surg. 2019;54(8):1711–4.
